# Oxysulfur and organosulfur toxins: the hidden mediators of the oral-systemic connection

**DOI:** 10.3389/fmicb.2026.1823575

**Published:** 2026-06-29

**Authors:** Giuseppe Pezzotti

**Affiliations:** 1 Biomedical Engineering Center, Kansai Medical University, Hirakata, Osaka, Japan; 2 International Center for Biomedical Industrial Promotion, Kansai Medical University, Hirakata, Osaka, Japan; 3 Department of Immunology, Graduate School of Medical Science, Kyoto Prefectural University of Medicine, Kyoto, Japan; 4 Department of Orthopedic Surgery, Tokyo Medical University, Shinjuku-ku, Tokyo, Japan; 5 Department of Molecular Science and Nanosystems, Ca’ Foscari University of Venice, Venice, Italy; 6 Biomarker Disease Laboratory, IRCCS San Camillo Hospital, Venice, Italy; 7 Department of Orthopaedic Surgery, Graduate School of Medicine, Mie University, Tsu, Mie, Japan; 8 Teikyo University Institute of Medical Mycology (TIMM), Graduate School of Medicine, Teikyo University, Itabashi-ku, Tokyo, Japan; 9 Department of Bacteriology, Graduate School of Biomedical and Health Sciences, Hiroshima University, Hiroshima, Japan

**Keywords:** hidden mediators, oral-systemic connection, oxysulfur/organosulfur toxins, *Porphyromonas gingivalis*, Raman spectroscopy, *Streptococcus mutans*

## Abstract

The oral cavity hosts complex microbial communities whose pathogenic members are implicated in systemic diseases, including cardiovascular, metabolic, and neurodegenerative disorders. While proteinaceous virulence factors and inflammatory pathways have been extensively studied, the contribution of microbial sulfur chemistry to the oral–systemic connection remains poorly understood. Here, we report Raman spectroscopic evidence that two major oral pathogens, *Streptococcus mutans* and *Porphyromonas gingivalis*, actively exploit oxysulfur/organosulfur molecules as part of their pathogenic arsenal. In the former bacterium, the protein referred to as Collagen binding mutans (Cnm) protein, exhibits a high density of organosulfur-related vibrational signatures, consistent with the presence of chemically reactive sulfur moieties that may promote extracellular matrix adhesion and oxidative stress modulation. In parallel, the latter bacterium selectively enriches its outer membrane vesicles with oxysulfur-associated molecular species, suggesting a vesicle-mediated delivery mechanism for sulfur-based toxins to host cells. These findings point to a convergent strategy whereby chemically aggressive organosulfur and oxysulfur compounds function as molecular effectors at host–pathogen interfaces. By framing sulfur chemistry as a functional dimension of microbial virulence, this study provides a new chemical perspective on the oral–systemic connection and highlights Raman spectroscopy as a powerful tool for uncovering noncanonical pathogenic mechanisms at the molecular scale.

## 1 Introduction

In recent scientific advancements, the concept of an oral–systemic connection has evolved from epidemiological association to mechanistic inquiry, revealing that chronic oral infections can exert far-reaching effects on distant organs (Barnett, 2006; Natarajan et al., 2025; Cirillo, 2025). Pathogens such as *Streptococcus mutans* (*S. mutans*) and *Porphyromonas gingivalis* (*P. gingivalis*) are now recognized not only as local drivers of dental caries and periodontitis, respectively, but also as contributors to cardiovascular disease (Bloch et al., 2024; Mei et al., 2020), metabolic dysregulation (Mei et al., 2020; Wang et al., 2022), and neurodegeneration (Huang et al., 2025; Dominy et al., 2019; Pezzotti et al., 2023; Hosoki et al., 2020). It should be noted at the outset that, as part of the normal oral microbiome, these two bacteria are harmless pathobionts. It is only after microbial dysbiosis occurs due to environmental changes that they can be referred to as pathogens. Despite a growing recognition of such pathogenic drifts, the molecular strategies that enable oral bacteria to transcend the anatomical boundaries of the oral cavity yet remain incompletely understood.

Traditionally, research on oral pathogen virulence has focused on proteases, adhesins, lipopolysaccharides, and host inflammatory cascades (Curtis et al., 2005; Santos et al., 2025; Lunar Silva and Cascales, 2021). While these factors are undoubtedly important, such frameworks tend to underrepresent the role of microbial chemical reactivity, particularly involving sulfur-containing compounds. Sulfur is unique among biologically relevant elements in its ability to access multiple oxidation states and to participate in redox reactions, nucleophilic attacks, and metal coordination (Doman et al., 2023; Hou et al., 2025). In microbial systems, sulfur chemistry is frequently associated with cytotoxicity, redox manipulation, and disruption of host signaling pathways. Yet, its involvement in oral pathogen–host interactions has received surprisingly little attention.

Recent advances in Raman spectroscopy have enabled the direct, label-free detection of chemically specific functional groups in intact biological systems (Pezzotti, 2021). Importantly, Raman spectroscopy is uniquely sensitive to sulfur-containing bonds, including S–S, C–S, and oxidized sulfur species, providing a powerful window into microbial sulfur chemistry at the molecular scale (Sato et al., 1985). Leveraging this capability, we investigated whether oral pathogens employ sulfur-based molecular strategies that are not readily captured by conventional biochemical assays. In *S. mutans*, the collagen-binding protein Cnm has emerged as a critical virulence factor implicated in systemic dissemination and vascular pathology. While Cnm is classically described in terms of its adhesive function (Nobbs, 2016), its detailed chemical composition and reactivity yet remain conspicuously underexplored. Our *in situ* Raman spectroscopic analyses revealed that Cnm is enriched in organosulfur-associated vibrational features and suggest a role as chemically active sulfur moieties (Pezzotti et al., 2026a). Organosulfur species may enhance collagen binding through covalent or quasi-covalent interactions, modulate local redox environments, or induce host tissue damage that facilitates bacterial persistence beyond the oral niche. In parallel, *P. gingivalis* is known to deploy outer membrane vesicles (OMVs) as long-range delivery systems for virulence factors (Gui et al., 2016). OMVs can easily penetrate host tissues, cross biological barriers, and directly interact with immune and neuronal cells (Pezzotti et al., 2023; Zhang et al., 2025). Our Raman data demonstrate that these vesicles are selectively loaded with oxysulfur-related molecular species, pointing to a deliberate vesicle-mediated export of sulfur-based toxins. This strategy may allow *P. gingivalis* to exert chemically crafted aggression strategy at sites distant from the primary infection, amplifying its systemic impact while minimizing direct bacterial exposure to host defenses.

Taken together, these observations suggest that *S. mutans* and *P. gingivalis*, despite their phylogenetic and ecological differences, share a convergent reliance on sulfur chemistry as a trigger for their virulence mechanisms. In this paper, we shall bring together our previously collected experimental evidences on Raman assessments of *S. mutans* and *P. gingivalis* bacteria, and newly propose a view according to which organosulfur/oxysulfur-containing compounds act as *molecular disruptors* at the oral–systemic interface, facilitating tissue invasion, immune modulation, and systemic dissemination. By integrating Raman spectroscopic evidence with current concepts of microbial pathogenicity, this work introduces sulfur chemistry as a previously underappreciated dimension of the oral–systemic connection and opens new avenues for diagnostics and therapeutic intervention.

## 2 Experimental procedures

### 2.1 Cell culture procedures

The human neuroblastoma SH-SY5Y cell line was purchased from ECACC.^1^ Cells were pre-cultured in RPMI1640 (Nacalai Tesque, Kyoto, Japan) supplemented with 10% heat-inactivated fetal bovine serum and 1% penicillin–streptomycin (Nacalai Tesque, Kyoto, Japan) in a 5% CO_2_ incubator. Cells were seeded at a density of 4 × 10^5^ cells/mL in 6-well glass-bottom plates (MatTek Corporation, Ashland, MA, USA), manually coated with Recombinant Human Laminin-511 E8 Fragment iMatrix-511 (Matrixome Inc., Osaka, Japan), and then incubated in a CO_2_ incubator at 37 °C overnight. After changing the culture medium to a new serum-free medium containing 1% penicillin–streptomycin either without or with *P. gingivalis* OMVs (concentration of 10 μg/mL), the cells were further incubated for 6 h to perform observation and analyses before and after exposure to *P. gingivalis* OMVs. All experiments were conducted on three separate dishes per each type of experiment (*n* = 3).

The Cell Count Reagent SF (WST-8; Nacalai Tesque, Kyoto, Japan) was used for colorimetric cell viability tests after 6 h cell exposure to OMVs. An additional SH-SY5Y cell sample was analyzed for comparison, which was combined with Pg-OMVs treated with an inhibitor for Arg- and Lys-gingipains (KYT-1; Peptide Institute, Inc., Ibaraki-shi, Osaka, Japan). Then, a water-soluble tetrazolium salt was applied to the culture supernatant and the absorbance at 490 nm was measured on a plate reader Infinite® F50 (Tecan Group Ltd., Männedorf, Switzerland). Cells were fixed with 4% paraformaldehyde (PFA) for 10 min at room temperature (RT) and washed with 0.1% triton X in phosphate-buffered saline (PBS); then, they were used for fluorescence microscopy experiments. Cell staining was performed using primary antibodies: Anti-Phosphorylated Tau (Clone: C5) and Anti-Human Amyloid *β* (1–42) antibodies (Immuno-Biological Laboratories Co, Ltd., Fujioka, Gunma, Japan) for 60 min at room temperature. After washing with wash-buffer, cells were incubated with an Alexa 594 Goat Anti-Mouse IgG or Alexa 594 Goat Anti-Rabbit IgG (Thermo Fisher Scientific, Waltham, MA, USA), Hoechst 33342 (Dojindo, Co., Ltd., Kumamoto, Japan) and Phalloidin Alexa 488 (Thermo Fisher Scientific, Waltham, MA, USA) for 60 min at room temperature in the dark. The stained cell cultures were then observed under a fluorescence microscope (BZX710; Keyence, Osaka, Japan).

### 2.2 Culture of *Streptococcus mutans* bacteria

The strain KSA153 was isolated from the oral cavity of a volunteer. This bacterial strain was primarily isolated as part of a previous study for which ethical approval was obtained. Cnm-positive (*Cnm*^(+)^) and cnm-lacking (*Cnm*^(−)^) bacterial strains were cultured in trypticase soy broth (Becton Dickinson Microbiology Systems, Cockeysville, MD, USA) or Trypticase soy agar (TSA) added with erythromycin (10 μg/mL) in 5% CO_2_ atmosphere at 37 °C; *cnm* single-deletion mutants (KSM153 *Δcnm*), kindly provided by Prof. M. Kawada-Matsuo at Hiroshima University, were constructed via overlapping extension polymerase chain reaction (PCR), as described in detail in a previous paper (Kawada-Matsuo et al., 2013).

A collagen binding assay was employed in a comparative experiment on *Cnm*^(+)^ and *Cnm*^(−)^ bacterial strains in order to check their adhesion characteristics on collagen IV. *S. mutans* strain suspensions were added to collagen-coated dishes and incubated at 37 °C for 2 h. The bacterial suspension was then removed, washed with PBS, and treated with 0.25% trypsin solution to detach the adherent bacteria.

### 2.3 Isolation and microscopy observation of *Porphyromonas gingivalis* OMVs

*Porphyromonas gingivalis* OMVs were prepared from *P. gingivalis* culture supernatant as described previously (Kariu et al., 2019). Briefly, *P. gingivalis* ATCC33277 was grown in BHI-broth supplemented with hemin and menadione under anaerobic conditions achieved using the Anaero Pack System. Ammonium sulfate-sedimented proteins in culture supernatant were collected by centrifugation and then dialyzed against 10 mM sodium phosphate buffer at pH 7.4. Protein concentration was then determined using the Protein Assay CBB Solution (Nacalai Tesque, Kyoto, Japan). Transmission electron microscopy (TEM) observation was performed using a JEM-2100 (JEOL, Tokyo, Japan) with an acceleration voltage of 80 keV. The *P. gingivalis* OMVs were negatively stained with 0.1% phosphotungstate. The dynamic light scattering (DLS) method was used for assessing the size distribution of *P. gingivalis* OMVs using a commercially available particle size analyzer (ELSZ-1000, Otsuka Electronics, Osaka, Japan) at 25 °C. The light source was a He-Ne laser with a wavelength of 630 nm set at an angle of 45°. Experimental data were analyzed using the algorithm provided by the manufacturer.

### 2.4 Raman spectroscopic analyses

*In situ* Raman experiments were conducted by means of a specially designed spectrometer (LabRAM HR800, Horiba/Jobin-Yvon, Kyoto, Japan) set in confocal mode. This equipment employs a holographic notch filter to concurrently provide high-efficiency and high-resolution spectral acquisitions. The wavelength of the incoming laser was either 532 or 785 nm with the laser source operating with a laser power of 20 and 70 mW, respectively. The spectral resolution of ~1 cm^−1^ was achieved upon analyzing the Raman scattered light by a double monochromator connected with an air-cooled charge-coupled device (CCD) detector (Andor DV420-OE322; 1,024 × 256 pixels); the grating used in the spectrometer had a resolution of 1800 gr/mm. The acquisition time of a single spectrum was typically 10 s. Three consecutive acquisitions were made at the same spot to minimize noise. The laser spot was ~2 μm as focused on the sample through a 50x optical lens. In collecting average spectra, sets of 10 spectra were collected on each sample at different locations over areas of ~2 mm^2^. The raw Raman spectra were first subjected to a baseline subtraction procedure as preliminary optimized and standardized according to the asymmetric least square method (He et al., 2014). After baseline subtraction average spectra were deconvoluted into series of Lorentzian-Gaussian sub-bands using commercial software (LabSpec 4.02, Horiba/Jobin-Yvon, Kyoto, Japan). In performing this deconvolutive procedure, a machine-learning approach was applied, which employed an in-house built automatic solver described in previous studies (Pezzotti, 2021).

Raman imaging was performed by means of a dedicated Raman device (RAMANtouch, Nanophoton Co., Minoo, Osaka, Japan) operating in microscopic measurement mode (50x lens; numerical aperture, NA = 0.9) with confocal imaging capability in two dimensions. This Raman spectroscope achieved high in-plane spatial resolution (i.e., 300 nm) upon exploiting a specially designed spectrograph with completely compensated aberration. A specially designed confocal optics also allowed for high spatial resolution (~670 nm) along the out-of-plane *z*-direction. This Raman microscope was also capable of ultra-fast simultaneous acquisition of up to 400 spectra, greatly reducing laser irradiation time and thus enabling compatibility of the Raman scanning with cells’ life. The excitation source was a 532 nm solid-state laser operating with a power of 10 mW at the sample surface; a 300 grating was used, which led to a spectral resolution of ~2 cm^−1^ (spectral pixel resolution of 0.3 cm^−1^/pixel) with an accuracy in laser spot spatial location of 100 nm. Raman hyperspectral images were generated by means of commercially available software (Raman Viewer, Nanophoton Co., Minoo, Osaka, Japan). Lateral displacements involved steps of 500 nm for the laser focal point on the samples.

## 3 Experimental results

### 3.1 Raman characterizations of *Streptococcus mutans* strains

**Figure 1:**
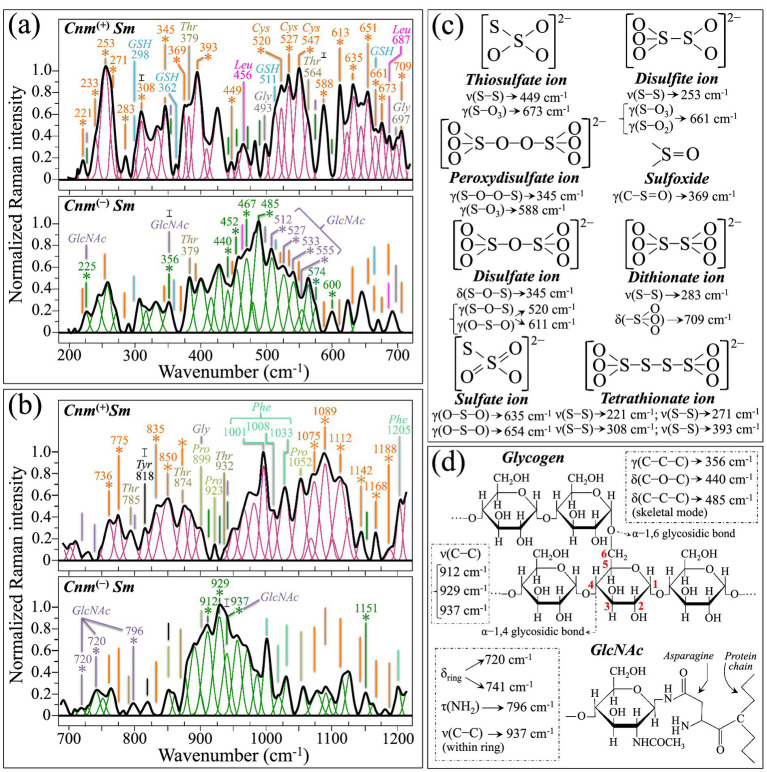
Average Raman spectra collected in the wavenumber intervals 200–700 cm^−1^ **(a)** and 700–1,200 cm^−1^ **(b)** on *Cnm*^(+)^ and *Cnm*^(−)^ *S. mutans* strains (cf. labels in inset) cultured in absence of sucrose; main signals from sulfur-containing molecules and signals characteristic of glycogen and N-acetylglucosamine molecules are reported in **(c)** and **(d)**, respectively. The shown spectra are the average of 10 spectra collected at different locations (over a total area of ~2 mm^2^); ± standard deviation bars are shown in correspondence of the 308, 588, and 356 cm^−1^ signals in **(a)** (S–S stretching in tetrathionate, S–O_3_ deformation in peroxydisulfate, and C–C–C deformation in glycogen, respectively); and, in correspondence of signals at 818 and 937 cm^−1^ in **(b)** (ring breathing in *Tyr* and C–C ring stretching in *GlcNAc*, respectively). The abbreviations *GSH*, *Leu*, *Gly*, *Thr*, *Tyr*, *Pro*, and *Phe* stand for glutathione, leucine, glycine, threonine, tyrosine, proline, and phenylalanine, respectively.

Figures 1a,b compare average Raman spectra collected in the wavenumber interval 200 ~ 700 cm^−1^ and 700 ~ 1,200 cm^−1^, respectively, on *Cnm*^(+)^ and *Cnm*^(−)^ *S. mutans* strains (cf. labels in inset) cultured in absence of sucrose (Pezzotti et al., 2026a). Each spectrum is the average of 20 spectra collected at different locations (over a total area of ~2 mm^2^). Since culturing *S. mutans* in absence of sucrose impedes the formation of biofilm, the spectra represent the molecular structure of bacterial cells only. The spectra were deconvoluted into a series of Gaussian-Lorentzian sub-bands with specific vibrational origins (cf. labels in inset). As can be seen, the spectral morphology in *Cnm*^(+)^ and *Cnm*^(−)^ *S. mutans* strains was very different over the entire wavenumber span investigated. Such differences unequivocally prove a significant divergence in molecular structure between the two strains. In the low-wavenumber interval (Figure 1a), the spectrum of the *Cnm*^(+)^ strain is rich in signals from sulfur-containing molecules, which are conspicuously lacking in the spectrum of the *Cnm*^(−)^ strain (Figure 1c). On the other hand, Raman signals characteristic of glycogen and N-acetylglucosamine (GlcNAc) molecules (Figure 1d) were clearly stronger in the spectrum of the *Cnm*^(−)^ strain, while conspicuously missing in that of the *Cnm*^(+)^ one. In the high-wavenumber zone (Figure 1b), signals from sulfur-containing molecules were confirmed to be stronger in the spectrum of the *Cnm*^(+)^ strain, a trend that was also common to the ring modes of phenylalanine (Phe). Conversely and consistently with the low-wavenumber spectra, C–C stretching signals at 912, 929, and 937 cm^−1^ from glucose and glycogen molecules were stronger in the *Cnm*^(−)^ strain. Main vibrational fingerprints for sulfur compounds, glycogen, and GlcNAc are reported in Figures 1c,d (cf. labels in inset) (Sato et al., 1985; Wiercigroch et al., 2017; She et al., 1974; Zhu et al., 2011).

### 3.2 *Porphyromonas gingivalis* OMVs characterizations and their interaction with neuroblastoma cells

**Figure 2:**
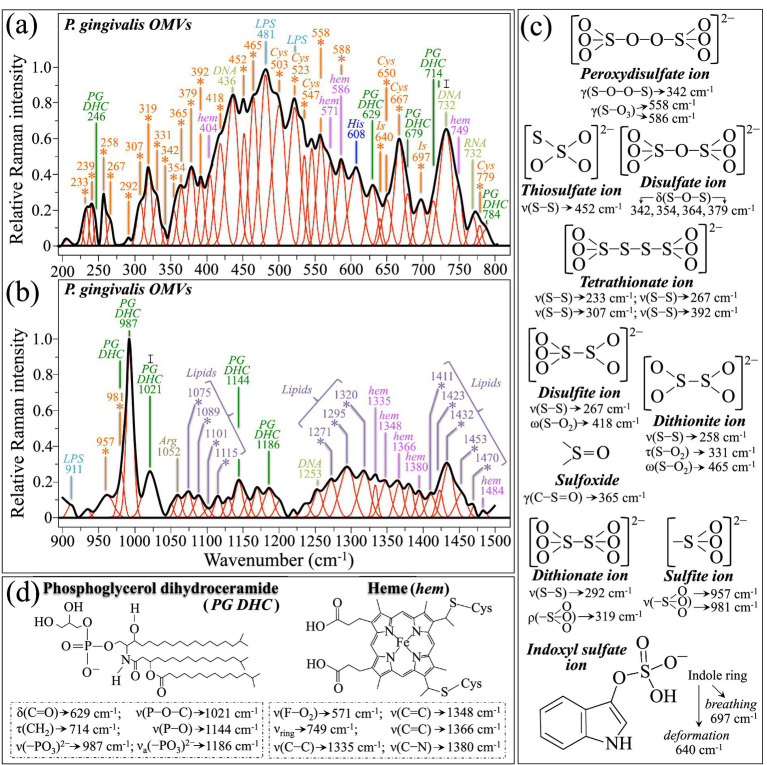
Average Raman spectra collected in the wavenumber intervals 200–800 cm^−1^ **(a)** and 900–1,500 cm^−1^ **(b)** on *P. gingivalis* OMVs; main signals from sulfur-containing molecules contained in the OMVs and signals characteristic of phosphoglycerol dihydroceramide (*PGDHC*) and heme (hem) molecules are reported in **(c)** and **(d)**, respectively. The shown spectra are the average of 20 spectra collected at different locations (over a total area of ~2 mm^2^). ± standard deviation bars are shown in correspondence of the 732 and 1,021 cm^−1^ signals in **(a)** and **(b)**, respectively (ring breathing in DNA adenine and P–O–C stretching in *PG DHC*, respectively). The abbreviations *LPS*, *His*, *Is*, and *Arg* stand for lipopolysaccharides, histidine, indoxyl sulfate, and arginine, respectively.

Figures 2a,b show high spectrally resolved (average) Raman spectra collected in the wavenumber intervals 200 ~ 800 cm^−1^ and 900 ~ 1,500 cm^−1^, respectively, on OMVs isolated from *P. gingivalis*. In the wavenumber zone 200 ~ 500 cm^−1^, a strong presence of S–S stretching and other S-bond-related signals was recorded, which arose from a number of distinct S–containing molecules (Figure 2c). Many of these signals were in common with the spectrum of *Cnm*^(+)^ strain, as shown in Figure 1a, although with slightly shifted wavenumbers given the different molecular contexts. The sulfur-containing molecules responsible for S–S signals could be identified as tetrathionate, disulfite, dithionate, disulfate, dithionite, and thiosulfate ions; disulfate and peroxydisulfate ions, which lack S–S signals, contributed spectral bands related to S–O–S bending and S–O–O–S deformation, respectively (cf. molecules and vibrational modes in Figure 2c) (Sato et al., 1985). Indoxyl sulfate (*Is*) also lacks strong signals in the low-wavenumber region, but presents a fingerprint signal from ring-breathing signal at 697 cm^−1^ (cf. label in inset to Figure 2a) (Elumalai et al., 2014), while an additional, but weaker, contribution at 640 cm^−1^ can be assigned to its ring deformation (Elumalai et al., 2014). Two bands at ~650 and 667 cm^−1^ are attributed to C–S stretching in cysteine (Lomont and Smith, 2022; Diaz Fleming et al., 2009; Shanmugasundaram and Puranik, 2009; Gong et al., 2009), while the signal at 503 cm^−1^ could be assigned to S–S stretching in cysteine-cysteine residues (Ozaki et al., 1987) and the bands at 545, 558 and 779 cm^−1^ are mainly contributed by S–S stretching in disulfide oxidized conformers (Ozaki et al., 1987; Biswas et al., 2007).

In addition to a low-wavenumber shoulder signal at 246 cm^−1^ (arising from C–C torsional mode), signals from the OMVs’ membrane consisting of three-aliphatic-chains-containing phosphoglycerol dihydroceramide (PGDHC) could mainly be found in the interval between 600 and 800 cm^−1^ (Figure 2d). PGDHC fingerprint signals were found at 629 cm^−1^ (overlapping contributions from out-of-plane C=O bending, N–C torsion, and CH_2_ rocking), at 679 cm^−1^ (O=CN and C–C–O deformations), and at 714 cm^−1^ (CH_2_ twisting and rocking). (Misra and Tandon, 2012; Zivanovic et al., 2021), while a fourth signal, located at 784 cm^−1^ (O–P–O asymmetric stretching) was mainly assigned to the PGDHC phosphoglycerol unit. The most prominent band in the 200 ~ 800 cm^−1^ spectral window appeared at 481 cm^−1^. This signal mainly arises from in-plane C–C–O bending in the glucose rings of lipopolysaccharides (LPS) and links to the glucose ring deformation signals detected at 523 cm^−1^ (Wiercigroch et al., 2017), which overlaps the S–S stretching signal from Cys-Cys residues. The periplasmic content of *P. gingivalis* OMVs includes a toxic complex of LPS, besides lipids, outer membrane proteins, and proteolytic enzymes (gingipains) (Okamura et al., 2021; Uemura et al., 2022; Zhou et al., 1998; Li et al., 2013; Le Sage et al., 2017). Additional bands are labeled in inset to Figure 2a. The presence of RNA was proved by the signal at 769 cm^−1^ band, which can be assigned to nucleobase-ribose ring (backbone) vibrations (Samuel et al., 2022). Proteomic analyses of *P. gingivalis* OMVs have shown that they carry several prominent His-coordinating heme-binding proteins, while gingipains are notably enriched in arginine (Arg) and lysine (Lys) residues (Shoji et al., 2010). In other words, *P. gingivalis* is a heme auxotroph that relies on host sources and its OMVs serve as vehicles for hemin-binding proteins that enable heme capture and transport (Shoji et al., 2010; Kosno et al., 2022). Accordingly, *P. gingivalis* OMVs functionally operate in micronutrient acquisition of heme molecules. Under resonance Raman conditions, which is the case here, heme-binding proteins exhibit strong signals attributable to Fe–ligand vibrational modes, which in turn correspond closely to three relatively strong signals observed here at 404, 571, and 586 cm^−1^ spectral features. The most chemically and biologically plausible assignments are (Spiro, 1985; Zhang et al., 2023): Fe–His or Fe–CO stretching (404 cm^−1^), Fe–O_2_ (571 cm^−1^), and Fe–ligand stretching in presence of thiolate or phenolate ligands (586 cm^−1^). In the spectrum recorded in the wavenumber interval 900 ~ 1,500 cm^−1^, the most prominent signal appeared at 987 cm^−1^ (cf. Figure 2b) and could be assigned to –PO_3_^2−^ stretching (Feng et al., 2022; Wegener et al., 1996). A low-wavenumber shoulder at 981 cm^−1^ has the same vibrational origin but this latter signal, together with the one at 957 cm^−1^, could overlap SO_3_^2−^ stretching mode in sulfite (cf. Figure 2c) (Kawada-Matsuo et al., 2013). The signal located at 1,021 cm^−1^ arises from P–OH and P–O–C stretching (Feng et al., 2022; Wegener et al., 1996), while additional phosphorus-related signal appears at 1,186 cm^−1^ (antisymmetric phosphate vibrations) and at 1,144 cm^−1^ (P=O stretching in glycerol phosphate) (Adichtchev et al., 2020; Hua et al., 2013). Additional heme signals are seen at 1,335 (porphyrin skeletal mode), 1,348 (C=C symmetric stretching in reduced pyrrole ring), 1,366 (C=C symmetric stretching in partially oxidized pyrrole ring), and 1,380 cm^−1^ (C–N stretching and in-plane skeletal deformation in pyrrole ring) (Hu et al., 1993). Finally, the all region 1,411 ~ 1,470 cm^−1^ is dominated by scissoring and bending of CH_2_/CH_3_ moieties in lipid chains (Czamara et al., 2015).

## 4 Discussion

### 4.1 Interpreting Raman molecular insight into *Streptococcus mutans* Cnm^(+)^ strain

**Figure 3:**
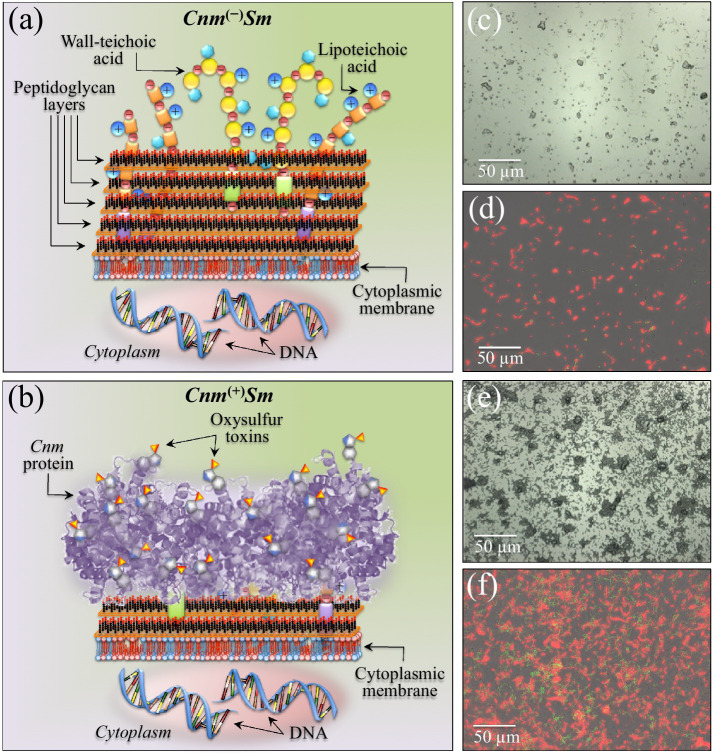
Schematic drafts summarizing the main structural differences between *Cnm*^(−)^ **(a)** and *Cnm*^(+)^ **(b)** strains, as deduced from Raman spectroscopic analyses; optical/fluorescence micrographs of *Cnm*^(−)^ and *Cnm*^(+)^ strains after deposition on and successive washing out from a collagen IV substrate are given in **(c,d)** and **(e,f)**, respectively.

Figures 3a,b schematically summarize the main structural differences between *Cnm*^(−)^ and *Cnm*^(+)^ strains, respectively, as revealed by Raman spectroscopic analyses. The formation of a highly disordered cnm protein layer in the *Cnm*^(+)^ strain occurred together with a significant simplification of the peptidoglycan wall-structure (including reduced amounts of teichoic and lipoteichoic moieties) as compared to the *Cnm*^(−)^ strain. The Cnm protein was replenished of toxins exhibiting sulfenic and sulfonic acid termini (cf. Figure 3b). Such a profound structural difference played a fundamental role when bacteria were placed on a collagen IV layer and then robustly washed out to test their adhesion capacity. As shown in Figures 3c,d (representing optical and fluorescence micrographs, respectively), only a limited number of *Cnm*^(−)^ bacteria was capable to resist the washing out flow and stick on the collagen IV layer. Conversely, bacteria from the *Cnm*^(+)^ strain tended to agglomerate and strongly adhered on the collagen IV surface despite the robust washing out flow (Figures 3e,f). This experimental evidence demonstrates the presence of a high-strength chemical bond that was exclusively established between the *Cnm*^(+)^ strain and the collagen substrate, thus supporting previously reported clinical findings on bacteria collected from a relative large number of patients (Pezzotti et al., 2026b; Pezzotti et al., 2026c).

**Figure 4:**
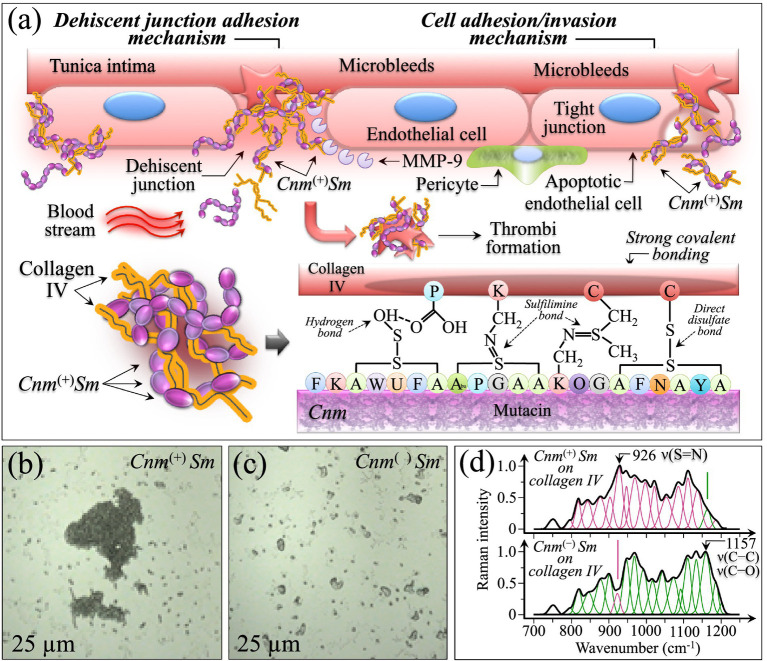
**(a)** Schematic draft showing how the *Cnm*^(+)^ strain likely exploits its superior adhesive capacity to either attach at dehiscent junctions or adhere/invade endothelial cells in order to form microbleeds that potentially become thrombi in ischemic strokes; in **(a)**, a scheme is also included of the possible chemical bonds between collagen IV and the bacteriocin cnm-mutacin. **(b,c)** Optical micrographs of *Cnm*^(+)^ and *Cnm*^(−)^ *S. mutans* strains deposited on a collagen IV substrate, and their respective Raman spectra in **(d)** (cf. labels in inset).

Figure 4a schematically shows how the *Cnm*^(+)^ strain likely exploits its adhesive capacity to attach at dehiscent junctions and/or to adhere to (and then to invade) endothelial cells in order to form microbleeds that potentially become thrombi in ischemic strokes. A draft of possible chemical bonds is also proposed in the same figure in an attempt to explain the high adhesion strength of the *Cnm*^(+)^ strain onto collagen IV. As depicted in this latter draft, besides S–S bonds, also strongly covalent sulfilimine bonds (S=N) could form. This suggestion obviously arises from the observation of a superior grip, as recorded for the *Cnm*^(+)^ strain only. However, the hypothesis of S=N bond formation is also supported by several structural circumstances already reported in literature: (i) *S. mutans* is known to produce enzymes that involve sulfur-containing compounds and are capable to produce mutacins in the oral environment (Balakrishnan et al., 2002); mutacins being lantibiotics that include amino acid residues involved in post-translational modifications (Merritt and Qi, 2012) (cf. mutacin structure in Figure 3a, as reported by Mota-Meira et al. (1997)); (ii) the collagen IV structure comprises highly homologous *α*-chains with amino-terminal domains rich in both cysteine and lysine, which could in principle form S=N bonds with mutacin-contained amino acids (cf. Figure 3a); and, (iii) following oxidation reactions at –SH thiol groups on the cnm protein surface, sulfenic or sulfinic acids could form, which are highly reactive species and could form S=N bonds with nearby nitrogen donors (e.g., lysine residues from collagen IV) (Ronsein et al., 2014).

In order to obtain an experimental proof that validates the hypothesis of sulfilimine bond formation at the Cnm/collagen IV interface, we collected *in situ* and compared the Raman spectra of *Cnm*^(+)^ and *Cnm*^(−)^ strains sticking on collagen IV after the washing out procedure (cf. samples shown in Figures 4b,c, respectively). These Raman spectra are given in Figure 4d (cf. labels in inset). As seen, despite some similarities, the Raman spectrum collected on different strains also presented some important differences. The band at 926 cm^−1^ appeared with intensity significantly enhanced in the *Cnm*^(+)^ strain as compared to the spectrum of *Cnm*^(−)^ one. On the other hand, the *Cnm*^(−)^ spectrum presented a significantly enhanced band at 1,157 cm^−1^ as compared to *Cnm*^(+)^ strain. According to literature (Evans, 1960; Arcoria et al., 1974; Muthuselvi et al., 2016), the band at 926 cm^−1^ could be assigned to S=N bond stretching, while the signal at 1,157 cm^−1^ is commonly associated with C–C and C–O stretching in carbohydrates (cf. also Figure 1b), including both cell-wall polysaccharides and extracellular polysaccharide components (Wiercigroch et al., 2017). Note that the relative intensity of the 1,157 cm^−1^ signal with respect to the C–C signals at 900 ~ 950 cm^−1^ as seen in Figure 4d is significantly stronger as compared to *Cnm*^(−)^ bacteria before exposure to collagen IV (cf. Figure 1b). This may reflect a compensatory, non-specific response of the *Cnm*^(−)^ strain to collagen IV exposure, characterized by increased polysaccharide production or surface carbohydrate presentation in the absence of a direct collagen binding. In contrast, the *Cnm*^(+)^ strain engages collagen IV through the Cnm adhesin, promoting a protein-dominated interface that promotes S=N bonds while diminishing the relative contribution of carbohydrate-associated Raman features. The preferential enhancement of the 1,157 and 926 cm^−1^ signals in *Cnm*^(−)^ and *Cnm*^(+)^ strains, respectively, is therefore consistent with their differential surface remodeling strategies, whereby *Cnm*^(−)^ cells rely more heavily on polysaccharide-mediated interactions, while *Cnm*^(+)^ cells form specific protein–protein bonds with collagen IV. This point will be further discussed in Section 4.3.

### 4.2 Interpreting Raman molecular insight into *Porphyromonas gingivalis* OMVs

**Figure 5:**
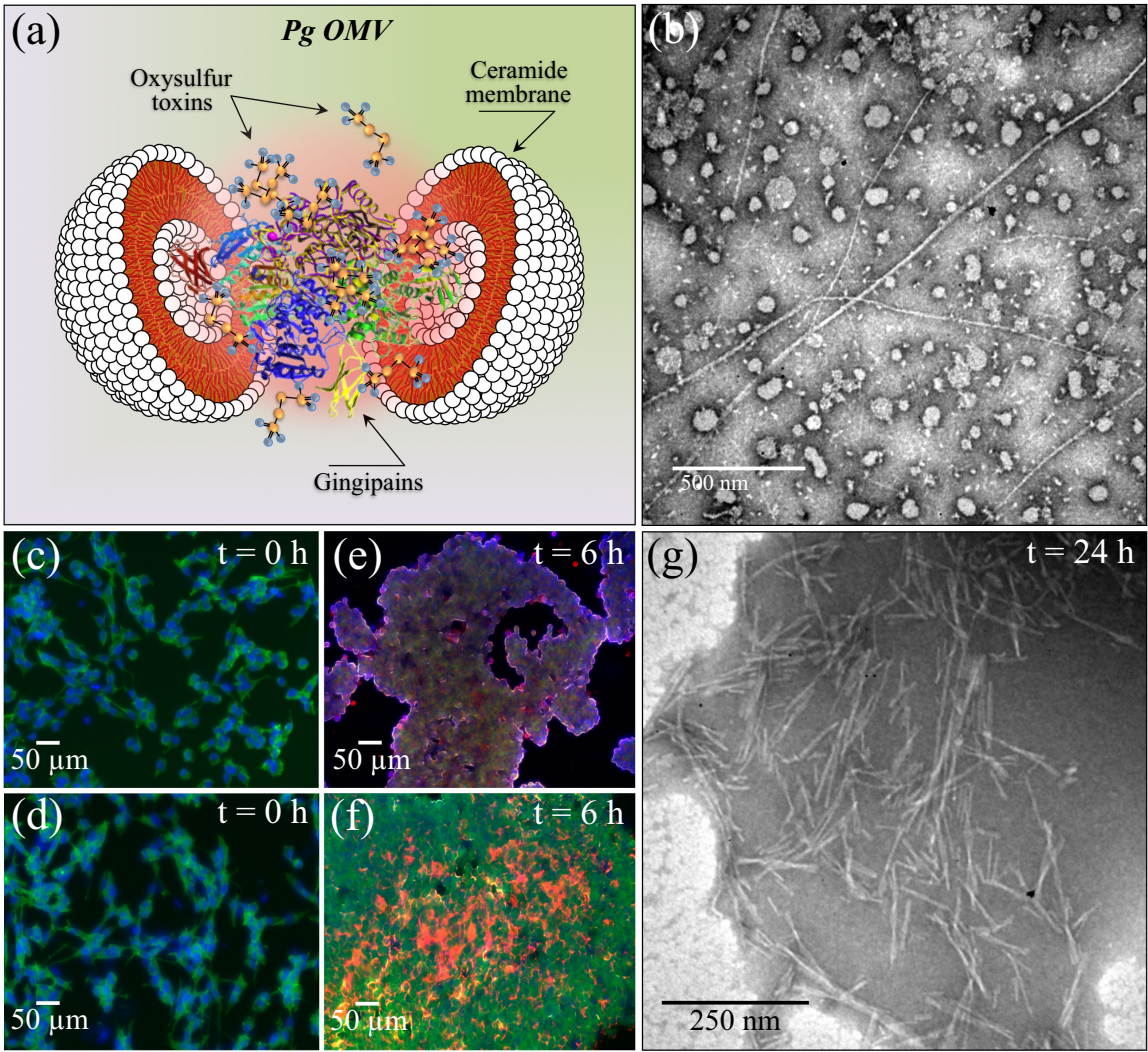
**(a)** Open up of a *P. gingivalis* OMV and spreading of its internal oxysulfur toxins and gingipains, and **(b)** TEM micrograph of *P. gingivalis* OMVs. In **(c,d)**, fluorescence micrographs of control SH-SY5Y cell cultures stained blue and green to visualize nuclei and actin filaments, respectively. Control cell cultures appear in a roundish shape and with a limited amount of neurites as expected in an undifferentiated state. The respective SH-SY5Y cell cultures are shown after exposure for 6 h to *P. gingivalis* OMVs and staining with anti-human amyloid β (1–42) antibody **(e)** and anti-phosphorylated Tau antibody **(f)**. In **(g)**, TEM image showing the formation of amyloid fibrils after 24 h exposure of SH-SY5Y cells to *P. gingivalis* OMVs.

**Figure 6:**
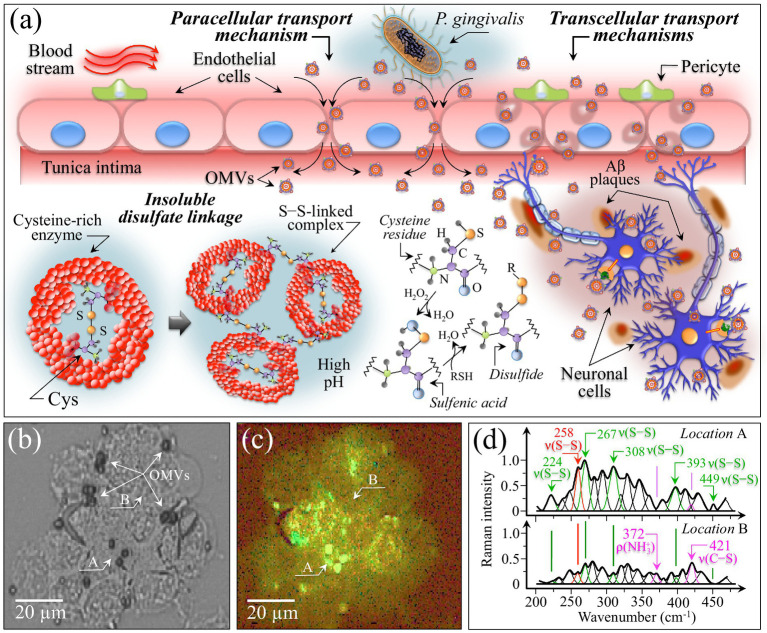
**(a)** Schematic draft of the possible routes by which *P. gingivalis* OMVs could penetrate the BBB, contaminate the brain tissue, and degrade neuronal cells; also in **(a)**, draft showing the formation of insoluble disulfate linkages at enzymatic sites and at oxidized cysteine sites, ultimately leading to plaque formation. Optical image **(b)** and hyperspectral Raman map **(c)** collected on a live neuronal cells culture contaminated with micrograms of *P. gingivalis* OMVs; the Raman map represents Raman intensities at two specific wavenumbers: 258 cm^−1^ (red) and 267 cm^−1^ (green), representing S–S bond stretching in dithionite and tetrathionate molecules, respectively. The overlap of the two colors is seen in yellow; **(d)** shows local Raman spectra extracted from the map at the two selected locations A and B (cf. labels in inset). The abbreviation Aβ stands for amyloid-β.

The analysis of the Raman spectrum of OMVs gives a clear structural view with respect to both their membrane configuration and toxic cargo (cf. Figures 2a,b). This analysis could help foreseeing the pathological effect on human health in the case such vesicles open up and spread around their toxic contents, as schematically shown in Figure 5a. Figure 5b shows the results of TEM observation on *P. gingivalis* OMVs (average diameter, *d_av_* = 35 nm). Upon adding OMVs in a concentration of 10 μg/mL to a culture of SH-SY5Y cells and incubating for 6 h, a dramatic morphological change could be observed. Fluorescence micrographs on cell cultures before and after exposure to OMVs are shown in Figures 5c–f. Control SH-SY5Y cell cultures were stained blue and green to visualize nuclei and actin filaments, respectively (Figures 5c,d). The respective SH-SY5Y cell cultures after exposure for 6 h to *P. gingivalis* OMVs were stained with Anti-Human Amyloid *β* (1–42) antibody (Figure 5e) and Anti-Phosphorylated Tau antibody (Figure 5f). Fluorescence micrographs collected after staining with primary antibodies revealed dramatic structural changes and the abundant presence of both Aβ and phosphorylated Tau peptides in OMVs-exposed cells. After 24 h, formation of Aβ fibrils was clearly observed (Figure 5g). The fluorescence microscopy data in Figures 5e,f give a vivid experimental confirmation that Aβ and phosphorylated Tau can form at an early stage of *P. gingivalis* OMVs’ contamination and without the need of a direct involvement of bacterial cells. Figure 6a schematically draws the possible routes by which *P. gingivalis* OMVs could penetrate the blood–brain barrier (BBB), contaminate the brain tissue, and degrade neuronal cells. Once *P. gingivalis* bacteria release OMVs into the blood stream, penetration can occur either by paracellular or transcellular transport mechanisms (cf. Figure 6a and the forthcoming Section 4.4). Formation of insoluble disulfate linkages at enzymatic sites and at oxidized cysteine sites could then ultimately lead to plaque formation, as observed in Figures 5e,f. In order to substantiate the molecular mechanisms behind plaque formation, we collected a hyperspectral Raman map on live neuronal cells contaminated by *P. gingivalis* OMVs under the same conditions followed for the experiments in Figures 5c–f. The results are shown in Figures 6b,c. Two specific wavenumbers were targeted: 258 cm^−1^ (depicted in red) and 267 cm^−1^ (depicted in green), which represent S–S bond stretching vibrations in dithionite and tetrathionate molecules, respectively (cf. Figure 2c) (Kawada-Matsuo et al., 2013). The overlap of the two colors is seen in yellow. These two wavenumbers were selected as representative for oxyanions and polythionates, respectively. As seen, the Raman maps visualize spots with high concentration of these two sulfur compounds not necessarily in correspondence of OMVs, but also in zones both adjacent to and relatively far from them, thus suggesting that sulfur toxins have been ejected and widely contaminated the cellular environment. Instructive in further analysis was a comparison between local spectra collected at high and low contaminated zones (cf. zones A and B, respectively, in Figures 6b,c), as shown in Figure 6d. This spectroscopic analysis showed enhanced intensity for all signals of tetrathionate ions at the highly contaminated location A, which instead conspicuously lacked two main cysteine bands at 372 cm^−1^ (rocking of NH_3_^+^ groups) and 421 cm^−1^ (C–S stretching) (Zhu et al., 2011) present in the spectrum collected at the low contaminated location B. This observation suggests the occurrence of a molecular mechanism of polysulfide bond formation at the expenses of cellular cysteine residues.

### 4.3 The sulfur-based persistence strategy of Cnm-positive *Streptococcus mutans* within the endothelial niche

In this study, we tested the hypothesis that the Cnm-positive *S. mutans* bacterium has shifted its functionality and utilizes sulfur chemistry to adhere to the endothelium and overcome the shear forces of blood flow. Driven by direct experimental and vibrational evidences, the key strength of this hypothesis resides in the fact that not a mere “sulfur metabolism” is invoked here, but evidence is provided that a specific oxysulfur chemical landscape is uniquely associated with the structure of Cnm-positive *S. mutans*. Moreover, evidence is also provided that collagen IV, namely, a compound that endothelial basement membrane is rich in, once subjected to oxidative stress during inflammation and exposed to peroxidases and reactive oxygen/nitrogen species, provides a chemically “permissive” endothelial enclave capable to hijack the endogenous crosslinking machinery. In experimental models of vascular endothelium, Cnm-positive strains demonstrated significantly increased adherence to and invasion of human coronary artery and umbilical vein endothelial cells compared with Cnm-negative or cnm-knockout strains (Abranches et al., 2011). Loss of the Cnm protein markedly reduced both adhesion and intracellular uptake, establishing active endothelial cell invasion as a Cnm-dependent process (Abranches et al., 2011; Mieher et al., 2025). Mechanistic studies indicated that the interactions between the Cnm protein and type IV collagen on endothelial surfaces stimulate host cell signaling pathways that involve small G protein regulators, thus inducing cytoskeletal rearrangements facilitating bacterial internalization rather than passive entrapment (Nomura et al., 2020). On the other hand, parallel clinical and translational research suggests a model wherein attachment at sites of endothelial junctional disruption (dehiscence) also contributes to vascular localization. According to this model, Cnm-positive *S. mutans* can adhere directly to denuded basement membrane exposed by endothelial tight junction breakdown, a process potentially exacerbated by inflammation and matrix metalloproteinase activity, which may precede or accompany invasion *in vivo* (Hosoki et al., 2022). In sum, according to so far published literature, while endothelial junction dehiscence provides a permissive niche for Cnm-mediated adherence to exposed collagenous surfaces, active invasion of endothelial cells via Cnm–collagen binding and host signaling induction has been indicated as the principal mechanism characterized in cell culture and animal models. Eventually, both pathways (cf. Figure 4a) synergize in pathological contexts such as infective endocarditis and cerebrovascular injury in bacteremia (Hosoki et al., 2020; Nomura et al., 2020).

The present Raman findings, while conceptually aligning with what is known about *S. mutans* contribution to the risk of stroke, also emphasize the role of sulfur in the biochemical processes underlying thrombous formation. Sulfur has primarily been indicated as a component of critical enzymes and amino acids that, via disulfide bond formation, regulate both coagulation cascade and platelet function (Chen and Hogg, 2006; Cho et al., 2008). The Raman spectroscopic detection of multiple organosulfur/oxysulfur anions (i.e., including thiosulfate, disulfite, dithionite, tetrathionate, peroxydisulfate, sulfate, and sulfoxide species) exclusively in the *Cnm*^(+)^ strain (cf. Figure 1a) provides clear evidence that Cnm expression can establish a highly specialized sulfur redox microenvironment at the bacterial surface. It is hypothesized here that such a chemically rich sulfur landscape plays a direct and active role in mediating high-affinity adhesion to collagen IV within the endothelial basement membrane. Moreover, collagen IV is uniquely susceptible to sulfur-driven chemistry due to its abundance of nucleophilic amide nitrogens, hydroxylysine residues, and methionine sulfur atoms, as well as its physiological incorporation of sulfilimine (S=N) crosslinks that stabilize the NC1 domain (cf. Figure 4a) (Roy and Gauld, 2024). Reactive sulfur species, particularly polythionates and peroxydisulfates, are capable of acting as electrophilic sulfur donors or redox mediators, enabling the formation of strong intermolecular interactions through S–O···H–N hydrogen bonding, thioether–amide charge-transfer complexes, or covalent sulfur–nitrogen linkages (Kolluru et al., 2020; Iciek et al., 2022). Among these possibilities, the formation of sulfilimine-like bonds between bacterial sulfur species and collagen IV amide nitrogens represents a particularly compelling mechanism, as sulfilimine covalent bonds are among the strongest known biological crosslinks and are already natively present in collagen IV architecture (cf. Figure 4a) (Roy and Gauld, 2024). The presence of lone pairs at nitrogen and sulfur atoms plays an important role in the biochemical reduction, which allows establishing a sulfenamide compound, as well as in oxidation reactions resulting in sulfoximine derivatives (Priya et al., 2025). Such an irreversible or quasi-irreversible anchoring, whose formation appears to be confirmed by the present *in vitro* Raman experiments (cf. Figures 4b–d), provides the molecular basis for persistent endothelial adhesion, bacterial aggregation, and subsequent thrombus nucleation under shear flow, thereby offering a chemically explicit pathway that links oral colonization by *Cnm*^(+)^ *S. mutans* bacteria to intravascular thrombosis and ischemic stroke. As shown in Figures 4b–d, our Raman study, for the first time, experimentally demonstrated the formation of S=N bond exclusively at the interface between a *Cnm*^(+)^ strain and collagen IV, thus proposing such a strong bond as the main responsible for the chemical mechanism ultimately leading to adhesion to the endothelium and, ultimately, to thrombus formation.

In summary, the selective presence of sulfur vibrational signatures in Cnm-expressing strains supports the notion that sulfur chemistry is not incidental but functionally integrated into the pathogenic adhesion machinery. Accordingly, the idea that *Cnm*^(+)^ *S. mutans* weaponizes sulfur chemistry to exploit the intrinsic sulfur–nitrogen crosslinking vulnerability of collagen IV is novel. Even if sulfilimine bonds are only partially formed, the combination of strong sulfur-mediated interactions could fully explain a number of the reported pathogenic outcomes.

### 4.4 The sulfur-based chemical weaponry of *Porphyromonas gingivalis* by redox re-engineering

Raman spectroscopic analysis of OMVs isolated from *P. gingivalis* revealed a striking enrichment of sulfur-based chemical species, including S–S stretching modes attributable to thiosulfate, disulfite, dithionite, and tetrathionate ions, S–O stretching modes associated with peroxydisulfate, disulfide, and sulfate species, and pronounced S=O stretching signatures characteristic of sulfoxides (cf. Figures 2a,b). We thus hypothesized, and showed some experimental evidences here, that this sulfur-rich chemical payload might constitute one key virulence mechanism by which *P. gingivalis* OMVs exert cytotoxic and immunomodulatory effects on macrophages, neutrophils, and competing microbial communities, while simultaneously promoting neurodegenerative pathology among neuronal cells. These sulfur species are redox-active and uniquely capable of inducing thiol oxidation, disulfide exchange, and sulfur-mediated crosslinking reactions in host proteins (Doman et al., 2023; de Diego et al., 2013). Consistent with this view, exposure of neuronal cell cultures to microgram quantities of OMVs resulted in a rapid cellular agglomeration into amyloid-*β*–like clusters within hours, strongly suggesting that the OMV-associated sulfur chemistry actively drives protein misfolding and aggregation (cf. Figures 5c–f). Based on the present Raman evidences, a particularly plausible mechanism appears to involve the formation of intermolecular disulfide (S–S) bridges between exposed cysteine residues in peptides or between neuronal surface enzymes and proteins (cf. Figures 6c,d), thereby stabilizing oligomeric nuclei and accelerating fibrillogenesis (cf. Figure 5g). Disulfide-locked oligomers are indeed known to be quite stable and neurotoxic (Sayre et al., 2008). Sulfoxide and polythionate species may further catalyze redox cycling, promoting repeated thiol oxidation and aggregation propagation. The detection of highly similar sulfur-related compounds in Alzheimer’s mouse models could be interpreted as supporting the pathological relevance of brain oxysulfur chemistry *in vivo* (Kinno et al., 2023). Recent clinical evidences also implicated dysregulated sulfur redox metabolism in human neurodegeneration. Reekes et al. (2023) have reported that plasma total sulfides, including bound persulfide/polysulfide and acid-labile sulfur pools, are significantly elevated in Alzheimer’s disease and related dementias, and correlate with cognitive decline, cortical thinning, white-matter disruption, and hippocampal atrophy, linking reactive sulfur species to brain structural degeneration. These sulfur pools comprise redox-active species capable of oxidizing protein thiols, destabilizing iron–sulfur clusters, and amplifying oxidative stress, particularly at the neurovascular interface. In parallel, *P. gingivalis* OMVs transport a package of sulfur-associated, thiol-dependent virulence factors, and deliver such chemically reactive moieties to host cells (cf. Figures 2a, 6c,d). The degenerative biochemical signatures detected by *in situ* Raman spectroscopy in neuronal cells exposed to such OMVs are therefore mechanistically concordant with findings on humans, as OMV-derived oxysulfur species represent a plausible exogenous source of reactive sulfur species capable of inducing the same redox imbalance and structural injury reflected by elevated plasma sulfurs. Collectively, the present data support a model in which bacterially derived oxysulfur stress contributes to systemic sulfur dysregulation and vascular dysfunction; their possible effect on neuronal degeneration could bridge peripheral sulfur signatures in humans with cellular neurotoxicity.

As an additional feature in this study, the ceramide-rich membrane composition of *P. gingivalis* OMVs, confirmed by specific Raman ceramide signatures (cf. Figure 2b), provides the physicochemical basis for their exceptional tissue penetration, including traversal of the blood–brain barrier, while also providing a rationale for their selective interaction with neuronal lipid microdomains. Collectively, our Raman findings support a mechanistic model in which *P. gingivalis* OMVs act as mobile, chemically aggressive vectors that disseminate toxic sulfur species systemically and disrupt innate immune cells; these effects could catalyze amyloid aggregation in neural tissue. As schematically shown in Figure 6a, their BBB penetration capacity positions OMVs as fundamental drivers of Alzheimer’s disease pathology within the oral–systemic axis. Emerging evidence indicates that the predominant mechanism by which *P. gingivalis* OMVs traverse the BBB involves engagement with host endothelial cells and exploitation of transcellular transport processes rather than an exclusively paracellular leakage (cf. Figure 6a) (Castro-Vargas et al., 2024). OMVs have been reported to interact with host cells via endocytic pathways including lipid-raft and caveolin-mediated internalization, consistent with the transcellular uptake mechanisms described for bacterial OMVs more broadly (Castro-Vargas et al., 2024; O’Donoghue and Krachler, 2016; Wang et al., 2023). However, in cerebral endothelial cells, *P. gingivalis* OMVs deliver potent virulence factors that degrade tight junction components (e.g., ZO-1 and occludin), which can contribute to increase BBB permeability; this observation supports a role for both transcytotic and paracellular influences on barrier integrity (Lei et al., 2023; Qiu et al., 2025; Zou et al., 2023). Mechanistic *in vitro* and *in vivo* studies of BBB models demonstrated that *P. gingivalis* infection enhances caveolae formation and caveolin-1 expression in brain microvascular endothelial cells, promotes internalization of bacterial components, and increases transcytotic flux without necessarily inducing gross tight junction disruption under certain conditions, implicating a major role for transcellular transport via caveolae-mediated transcytosis regulated by the Mfsd2a/Caveolin-1 axis (Lei et al., 2023; Mekata et al., 2024; Andreone et al., 2017). This transcellular pathway allows OMVs, carrying diverse lipid and protein cargos, to be internalized at the luminal surface, trafficked intracellularly, and released at the abluminal surface, effectively crossing the endothelial cytoplasm. Concurrently, OMV-associated proteases can compromise tight junctions and favor paracellular passage, particularly under inflammatory states or high vesicle burdens, suggesting that paracellular movement may contribute under conditions of barrier disruption (Lei et al., 2023; Qiu et al., 2025; Zou et al., 2023). Once penetrated within the brain tissue the OMVs release their sulfur toxins (cf. Figure 5a), promoting the formation S − S linked enzymatic complexes as well as triggering sulfur oxidation whose products support intermolecular di- and polysulfide crosslinking and lead to irreversible cell–cell aggregation (cf. Figures 6b–d).

The original contribution of the present Raman study regards the bacterium pathogenic footprint; the sulfur chemistry revealed by Raman spectroscopy in *P. gingivalis* OMVs suggests a highly evolved survival strategy that relies on chemical delegation rather than intrinsic cellular robustness. Unlike fast-growing or metabolically dominant bacteria, *P. gingivalis* is a slow-growing, oxygen-sensitive organism that is poorly equipped for direct confrontation with host immune defenses or microbial competitors (How et al., 2016; Afzoon et al., 2023). In such context, the enrichment of OMVs with a broad spectrum of oxysulfur species points to an intentional exploitation of sulfur redox chemistry as an extracellular weapon system. Rather than passively recycling environmental sulfur metabolites, these findings support a model in which *P. gingivalis* actively transforms available sulfur sources into chemically aggressive species that are sequestered within OMVs and deployed remotely. This strategy allows the bacterium to induce oxidative stress, thiol oxidation, protein crosslinking, and immune cell dysfunction at a distance, without exposing its own fragile cellular machinery to the same chemical hazards. OMVs thus function as autonomous chemical effectors, capable of attacking macrophages, neutrophils, competing bacteria, and even neuronal tissue, as evidenced by their ability to rapidly induce amyloid-*β* aggregation. In this framework, *P. gingivalis* should not be viewed as intrinsically strong but rather as strategically sophisticated or “chemically clever,” outsourcing virulence to sulfur-laden vesicles that confer persistence, immune evasion, and systemic pathogenic reach. Sulfur chemistry therefore emerges not as a metabolic byproduct but as a central organizing principle of *P. gingivalis* pathogenicity, with OMVs acting as mobile, self-contained units of chemical warfare within the oral–systemic axis.

### 4.5 The dichotomy of growth-inhibitor sulfur as a virulence enhancer

The apparent dichotomy between the antimicrobial effects of sulfur-containing compounds and the sulfur-dependent virulence of *S. mutans* and *P. gingivalis* can be explained by considering the chemical form, concentration, localization, and metabolic utilization of sulfur within the bacterial microenvironment. Sulfur is simultaneously an essential nutrient and a potential toxicant, and oral pathogens appear to exploit this duality in a highly regulated manner. Both *S. mutans* and *P. gingivalis* require sulfur for the synthesis of sulfur-containing amino acids (i.e., cysteine and methionine), iron–sulfur proteins, redox-active enzymes, and virulence-associated surface structures. In the oral cavity, sulfur can be acquired from host proteins, peptides, glycoproteins, glutathione, and degraded cellular debris generated during inflammation. In such context, periodontal bleeding and tissue degradation create particularly sulfur-rich ecological niches (Sedghi et al., 2021).

In the case of *Cnm*^(+)^ *S. mutans* strains, sulfur-containing residues within adhesins and associated extracellular proteins may stabilize protein conformation through disulfide bonding and enhance adhesion to collagen-rich substrates. Because Cnm-mediated colonization favors tissue invasion and persistence, sulfur assimilation may indirectly support virulence by promoting maturation and structural integrity of adhesin complexes. Moreover, oxidative stress in inflamed tissues likely selects for sulfur-based redox buffering systems, including thiol-containing molecules that protect bacterial proteins from oxidation.

In *P. gingivalis*, sulfur metabolism is even more central to pathogenicity. This organism is strongly proteolytic and obtains sulfur largely through degradation of host proteins rich in cysteine and methionine. The bacterium converts these substrates into volatile sulfur compounds such as hydrogen sulfide and methyl mercaptan, which are cytotoxic to epithelial and immune cells. Moreover, the OMVs released by *P. gingivalis* can accumulate sulfur-containing metabolites, proteases, hemin-associated compounds, and oxidized lipids, thereby functioning as concentrated nanocarriers of virulence factors. Sulfur compounds within OMVs are known to contribute to redox modulation, epithelial damage, immune dysregulation, and anaerobic niche maintenance (Okamura et al., 2021). Controlled intracellular sulfur acquisition and metabolism thus provide these pathogens with essential biochemical resources that enhance adhesion, redox homeostasis, biofilm formation, tissue invasion, and toxin production.

On the other hand, exogenous sulfur-reactive molecules at high concentration can disrupt bacterial metabolism, induce oxidative stress, or damage membranes, thereby suppressing growth (Cowman et al., 1983; Kandalam et al., 2018). Thus, sulfur compounds become inhibitory or beneficial depending on context. In essence, both *S. mutans* and *P. gingivalis* have evolved mechanisms to transform sulfur from an environmental stressor into a strategic virulence asset.

### 4.6 Similarities and differences in sulfur-based chemical weaponries of bacteria

This study shows how cnm-positive *S. mutans* and *P. gingivalis* both exploit sulfur chemistry as a covert virulence strategy but, while the former subtly leverages sulfur-dependent redox and stress-tolerance mechanisms to persist and invade beyond the oral niche, the latter weaponizes sulfur metabolism in order to generate toxic and tissue damaging byproducts. The use of sulfur chemistry as intermediates relies both on organosulfur and oxysulfurs.

*S. mutans* exploits sulfur-containing compounds such as cysteine and methionine to support its oxidative stress resistance, and biofilm development within dental plaque. Sulfur metabolism contributes to maintaining cellular redox balance and enhances the bacterium’s persistence in the acidic oral environment. These adaptations strengthen biofilm stability and adherence to tooth surfaces, thereby facilitating prolonged acid production and enamel demineralization, which ultimately promotes the development of dental caries. It is newly shown here that *Cnm*^(+)^ *S. mutans* strains can mainly exploit organosulfur moieties, which are more functional to the formation of strong S=N covalent bonds, although sulfilimine bond formation should require strong oxidants generated by host peroxidasin (via halide-dependent radical chemistry). *S. mutans* is thus unlikely to catalyze this reaction autonomously; instead, its sulfur-containing motifs may exploit transient host-derived radicals/enzymes in inflamed tissues to stabilize attachment. In other words, the bacterium leverages host organosulfur chemistry and oxidative microenvironments, rather than scavenging or producing the full enzymatic machinery itself, in order to reinforce collagen-targeted adhesion. *In situ* Raman spectroscopic assessments on live *S. mutans* bacteria proved how organosulfur compounds, exclusively in cnm-positive *S. mutans*, could form covalent S=N bonds that are functional in enhancing endothelial adhesion (cf. Figure 3). Note that covalent sulfilimine (–S=N–) crosslinks structurally reinforce the basement membrane network of collagen IV through a unique organosulfur chemistry linking methionine sulfur to hydroxylysine nitrogen (Vanacore et al., 2009). Therefore, the cnm-positive *S. mutans* actually mimicks a mechanism already existent in the collagen structure. Upon exploiting its inherent sulfur-rich binding motifs, it produces adhesins for targeted attachment to collagen-rich tissues (cf. proposed links between mutacin structure and collagen IV in Figure 3a) (Nishi et al., 2023).

On the other hand, *P. gingivalis* exploits sulfur chemistry by metabolizing sulfur-containing amino acids (i.e., cysteine and methionine) to generate volatile sulfur compounds such as hydrogen sulfide and methyl mercaptan. These metabolites contribute to tissue toxicity, disruption of epithelial barriers, and modulation of host immune responses within the periodontal pocket. Sulfur metabolism also supports anaerobic growth, oxidative stress tolerance, and biofilm persistence of *P. gingivalis* in subgingival environments. Collectively, these effects promote chronic inflammation, connective tissue destruction, and alveolar bone resorption, thereby driving the progression of periodontitis. New insights from Raman spectroscopy show that *P. gingivalis* also relies on oxysulfur molecules in order to enhance the virulence of its OMVs’ cargo. This selective choice occurs because the S–O bonds are easily broken to form a stronger S–S link and can thus “bridge” the gap between two thiols more rapidly than stable organosulfur compounds. When bridging cysteine residues to form disulfide (S–S) bonds, oxysulfur compounds are the most effective “bridging agents” or intermediates, because they more readily activate the sulfur atom for nucleophilic attack. Our Raman data proved that *P. gingivalis* indeed utilizes sulfenic acids as effective initiators for the formation of S–S bonds between cysteines (cf. Figure 6a). *In situ* Raman assessments on neuronal cells exposed to OMVs indeed showed clear enhancements of fingerprint signals from specific S–S bonds in disulfite, thiosulfate, and tetrathionate ions in response to a substantial reduction of cysteine fingerprint signals (cf. Figure 6d).

## 5 Conclusion

This work proposes an unified pathogenic paradigm centered on sulfur chemistry as a hidden, mobile vector of the oral systemic connection. The employment/deployment of oxysulfur and organosulfur species are identified through Raman spectroscopic analyses as a unifying and previously underappreciated mechanism underpinning the systemic pathogenicity of two major oral bacteria, Cnm-positive *S. mutans* and *P. gingivalis*. Although these organisms adopt distinct biological strategies, they converge on a common chemical logic: the exploitation of reactive sulfur species to extend their pathogenic influence far beyond the oral cavity. In Cnm-positive *S. mutans*, oxysulfur-rich microenvironments appeared to be tightly associated with the Cnm adhesin, enabling exceptionally strong and persistent anchoring to collagen IV within the endothelial basement membrane. In contrast, *P. gingivalis*, acting as a “chemical engineer,” scavenges host sulfur compounds, chemically reshapes them into more reactive forms, and packages them for targeted delivery. Externalizing its sulfur chemistry into ceramide-rich outer membrane vesicles, it creates OMVs, which function as autonomous pathogenic units capable of penetrating host tissues, disabling immune cells, disrupting microbial competitors, and catalyzing amyloid-*β* aggregation in neuronal environments.

Despite these differing modes of action, i.e., localized endothelial integration vs. long-range vesicle-mediated dissemination, both pathogens rely on a shared arsenal of sulfur toxins whose redox activity, bond-forming capacity, and chemical persistence enable durable host manipulation. The detection of comparable sulfur vibrational signatures in diseased human tissues could further support the *in vivo* relevance of this chemistry and suggests that oxysulfur species represent a hidden but powerful vector linking oral dysbiosis to systemic pathologies, including cardiovascular disease and neurodegeneration. Collectively, the present findings argue for a paradigm shift in the understanding of the oral-systemic connection, in which sulfur chemistry is recognized not as a metabolic byproduct but as a central, mobile, and chemically aggressive driver of chronic disease. The present study is yet based on *in vitro* experiments and will require further *in vivo* verifications in order to definitely confirm that *S. mutans* and *P. gingivalis* use organosulfur and oxysulfur compounds to induce virulence factors which can lead to cardiovascular and neurodegenerative diseases, respectively. In the case of *P. gingivalis* OMVs, additional *in vitro* experiments should be addressed to differentiated neuronal cell lineages. However, recognizing oxysulfur and organosulfur species as functional pathogenic mediators opens new avenues for diagnostics, preventive strategies, and therapeutic interventions aimed at intercepting sulfur-driven molecular interactions before irreversible systemic damage occurs.

## Data Availability

The original contributions presented in the study are included in the article, further inquiries can be directed to the corresponding author.

## Footnotes

1
https://www.culturecollections.org.uk/nop/product/sh-sy5y
